# Is the effect of non-invasive ventilation on survival in amyotrophic lateral sclerosis age-dependent?

**DOI:** 10.1186/1472-684X-12-23

**Published:** 2013-05-24

**Authors:** Waltteri Siirala, Riku Aantaa, Klaus T Olkkola, Tarja Saaresranta, Arno Vuori

**Affiliations:** 1 Department of Anaesthesiology, Intensive Care, Emergency Care and Pain Medicine, Turku University Hospital and University of Turku, Kiinamyllynkatu 4-8, FI-20520 Turku 52, Finland; 2 Division of Medicine, Department of Pulmonary Diseases, Turku University Hospital, Hämeentie 11, FI-20520 Turku 52, Finland

**Keywords:** Amyotrophic lateral sclerosis, Survival, Non-invasive ventilation

## Abstract

**Background:**

Hypoventilation due to respiratory muscle atrophy is the most common cause of death as a result of amyotrophic lateral sclerosis (ALS). Patients aged over 65 years and presenting bulbar symptoms are likely to have a poorer prognosis. The aim of the study was to assess the possible impact of age and treatment with non-invasive ventilation (NIV) on survival in ALS. Based on evidence from earlier studies, it was hypothesized that NIV increases rates of survival regardless of age.

**Methods:**

Eighty-four patients diagnosed with ALS were followed up on from January 2001 to June 2012. These patients were retrospectively divided into two groups according to their age at the time of diagnosis: Group 1 comprised patients aged ≤ 65 years while Group 2 comprised those aged > 65 years. Each group included 42 patients. NIV was tolerated by 23 patients in Group 1 and 18 patients in Group 2. Survival was measured in months from the date of diagnosis.

**Results:**

The median age in Group 1 was 59 years (range 49 – 65) and 76 years in Group 2 (range 66 – 85). Among patients in Group 1 there was no difference in probability of survival between the NIV users and non-users (Hazard Ratio = 0.88, 95% CI 0.44 – 1.77, p = 0.7). NIV users in Group 2 survived longer than those following conventional treatment (Hazard Ratio = 0.25, CI 95% 0.11 – 0.55, p <0.001). ALS patients in Group 2 who did not use NIV had a 4-fold higher risk for death compared with NIV users.

**Conclusions:**

This retrospective study found that NIV use was associated with improved survival outcomes in ALS patients older than 65 years. Further studies in larger patient populations are warranted to determine which factors modify survival outcomes in ALS.

## Background

Amyotrophic lateral sclerosis (ALS) is a neuromuscular disease characterized by progressive muscular atrophy throughout the body. Hypoventilation due to respiratory muscle atrophy is the most common cause of death in advanced ALS cases [1,2]. The presence of bulbar symptoms at the time of diagnosis and advanced age (>65 years) have been associated with poorer survival outcomes [3]. No curative treatment is currently available and intervention is always palliative [4-7]. Riluzole, a tedrotoxin-sensitive sodium channel blocker, is the only existing treatment and can delay disease progression with few months [8,9].

Non-invasive ventilation (NIV) can relieve dyspnoea, increase quality of life, and improve survival outcomes; particularly among ALS patients without severe bulbar symptoms [10-13]. Several guidelines currently recommend the use of NIV as palliative treatment for ALS [4-7]. The effect of age has not been addressed in previous studies assessing the impact of NIV on survival outcomes [10,12,13]. Therefore, the effect of NIV on survival was compared in patients aged 65 years and older of age at the time of diagnosis, designated as Group 1 and Group 2 respectively. Based on previous studies [10,12,13] it was hypothesised that NIV users would experience improved survival outcomes in both age groups when compared with patients who declined NIV were unable to tolerate treatment.

## Methods

### Design

A registry-based retrospective cohort study was undertaken, covering the period January 2001 to June 2012, on 91 patients fulfilling the El Escorial World Federation criteria for probable or definitive ALS [14]. Once a positive diagnosis was made by a neurologist, patients were referred to a specialist to evaluate their suitability for NIV. All patients were systematically followed up at intervals of 3 to 6 months, until the date of death or June 1 2012, when the follow-up period ended. NIV and other palliative treatments were offered to all the patients. Six patients showed survival over ten years and this finding was quite consistent with earlier studies which have been shown that 5 to 10% of ALS patients will survive over ten years [15]. Two of these six patients (age 54 and 79 years) used NIV while the rest of the patients (age range 49 – 63 years) refused NIV. Because of possible bias resulting from small number of patients with slow disease progression, these cases were excluded. In addition, one patient was excluded because of commencement of NIV before the time of diagnosis for treatment of respiratory insufficiency due to pulmonary embolism. Because of current clinical trials legislation in Finland, patient consent is not required for register studies. Patient consent was therefore not obtained in this instance. The study protocol was approved by the Ethics Committee of the Hospital District of South-West Finland.

**Figure 1:**
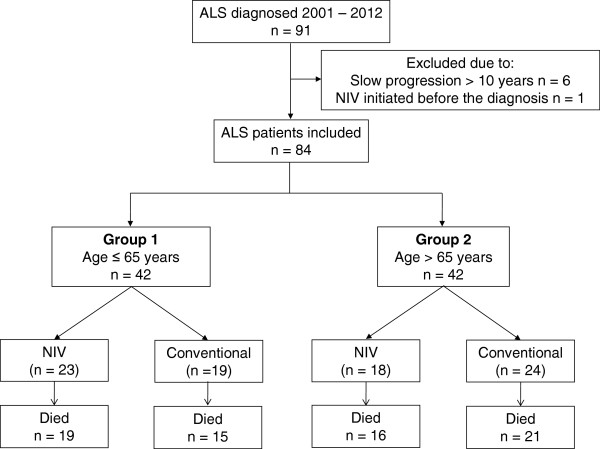
Flowchart of the study.

Altogether, 84 patients were included and retrospectively divided into two groups based on their age at the time of the diagnosis: Group 1 (age ≤ 65 years) and Group 2 (age > 65 years). Both groups were then subdivided further based on patients´ ability to tolerate the NIV. These were designated as the NIV Group and the Conventional Group, as presented in Figure 1.

Patients’ age, gender, self-reported time from first neurological symptoms to diagnosis (months), use of percutaneous endoscopic gastrostomy (PEG), use of NIV, use of riluzole and survival time from diagnosis were recorded. The presence of any cardiovascular comorbidity was adjusted for, because it may have modified survival outcomes. This was operationalized as a binary variable. A dichotomous variable was generated to categorize cases according to their initial symptoms and disease history, which were classified as either bulbar onset (difficulties in facial function or swallowing as an initial symptom) or spinal onset (voluntary muscle fatigue as initial symptoms). Patients presenting with both bulbar and spinal symptoms were classified as bulbar onset cases.

### Ventilatory support

All patients received information regarding hypoventilation treatment and the possibility of participating in the NIV trial. When hypoventilation occurred, suitability for NIV was assessed by a pulmonologist and an anaesthesiologist. The primary criteria for recommending for NIV were an increase in the partial pressure of carbon dioxide (pCO_2_) to over 5.5 kPa, or a decrease in the partial pressure of oxygen pO_2_ to below 10 kPa, measured by a morning arterial blood gas sample. Additional measurements included dyspnoea at rest, forced vital capacity (FVC), peak cough flow (PCF), maximum inspiratory mouth pressure (MIP), maximum expiratory mouth pressure (MEP), and sniff nasal pressure (SNP); all of which are considered secondary criteria for NIV diagnosis. These additional measurements were not always taken at the time of NIV initiation. Therefore, only pCO_2_ and pO_2_ measurements were reported, which were available for all patients. The final decision was based on each patient’s willingness to undergo NIV treatment, regardless of observed dyspnoea or an elevated morning pCO_2_.

NIV was given using a pressure-assisted ventilator (VPAP III ST®, ResMed, Bella Vista, Australia). The average weekly duration of NIV use was collected using the device’s in-built counter, normally at 3-month intervals. Patients undergoing NIV less than 4 hours per day at the last control visit, timed one week to 3 months prior to death, were considered NIV-intolerant and were allocated to the Conventional Group.

### Statistical analyses

The results are given as mean with 95% confidence intervals if not otherwise stated. Chi-square tests were used to compare discrete variables between the groups. Time (in months) from the onset of the symptoms until diagnosis was analysed using a Mann–Whitney *U* test. Comparison of the mean arterial pCO_2_ and pO_2_ at the moment of NIV initiation and the mean daily use of NIV was performed using a Student’s *t*-test. Survival time was measured in months from diagnosis until death or June 2012, when the follow-up ended. The interactions of age and NIV use with survival were assessed using a Cox regression. Survival curves were analysed using the Kaplan-Meier method and the Log-Rank Test. Proportional hazard assumptions were evaluated using Kaplan-Meier plots, with p < 0.05 denoting statistically significant difference. All analyses were carried out using SAS System version 9.2 for Windows (SAS Institute Inc., Cary, NC).

## Results

A total of 84 patients (37 male, 47 female) were included in this study. The median age in Group 1 was 59 years (range 49 – 65) and 76 years (66 – 85) in Group 2. NIV was tolerated by 23 subjects in Group 1 and 18 in Group 2. There were no statistically significant differences in any of the patient characteristics measured at baseline (see Table 1). Mean arterial pCO_2_ at the time of NIV initiation was 6.3 (SD = 1.5) kPa in the Group 1 NIV subgroup and 6.5 kPa (SD = 1.3, p = 0.7) in the Group 2 NIV subgroup. Mean arterial pO_2_ readings at the time of NIV initiation were 10.9 kPa (SD = 2.2) and 9.7 kPa (SD = 1.5, p = 0.1) for the Group 1 and Group 2 NIV subgroups, respectively. The mean daily duration of NIV at the last control visit prior to the death 17 h (SD = 7) in Group 1 and 14 h (SD = 6) (p = 0.2).

**Table 1:** Patients characteristics

	**Group 1 (age ≤ 65 years)**				**Group 2 (age > 65 years)**			
	**NIV (n = 23)**	**Conventional (n = 19)**	**p value**	**Number of total**	**NIV (n = 18)**	**Conventional (n = 24)**	**p value**	**Number of total**
Male / Female	10 / 13	10 / 9	0.6	20 / 22	9 / 9	8 / 16	0.3	17 / 25
Bulbar onset, number of patients (%)	9 (39%)	10 (53%)	0.4	19 / 42	5 (28%)	13 (54%)	0.1	18 / 42
Use of PEG, number of patients (%)	13 (57%)	10 (53%)	0.8	23 / 42	8 (44%)	10 (42%)	0.9	18 / 42
Use of riluzole, number of patients (%)	10 (43%)	11 (58%)	0.4	21 / 42	7 (39%)	5 (21%)	0.2	12 / 42
Hypertension or other cardiovascular diseases, number of patients (%)	8 (35%)	3 (16%)	0.3*	11 / 42	12 (67%)	11 (46%)	0.2	23 / 42
Median time from onset of symptoms until diagnosis, months (range)	12 (2 – 36)	10 (3 – 24)	0.5**		12 (7 – 60)	12 (1 – 54)	0.8**	
Median age at diagnosis, years (range)	61 (49 – 65)	58 (49 – 65)	0.3**		76 (66 – 85)	77 (66 – 84)	0.7**	

NIV = non-invasive ventilation, Conventional = no assisted ventilation, PEG = percutaneous endoscopic gastrostomy. The differences between the two groups were assessed using the Chi square test, except for number of patients having hypertension or other cardiovascular diseases, which was assessed with Fisher’s exact test*. The values for median age at diagnosis and time from onset of symptoms until diagnosis were compared with the Mann Whitney *U* test**. P values < 0.05 were considered statistically significant.

**Figure 2:**
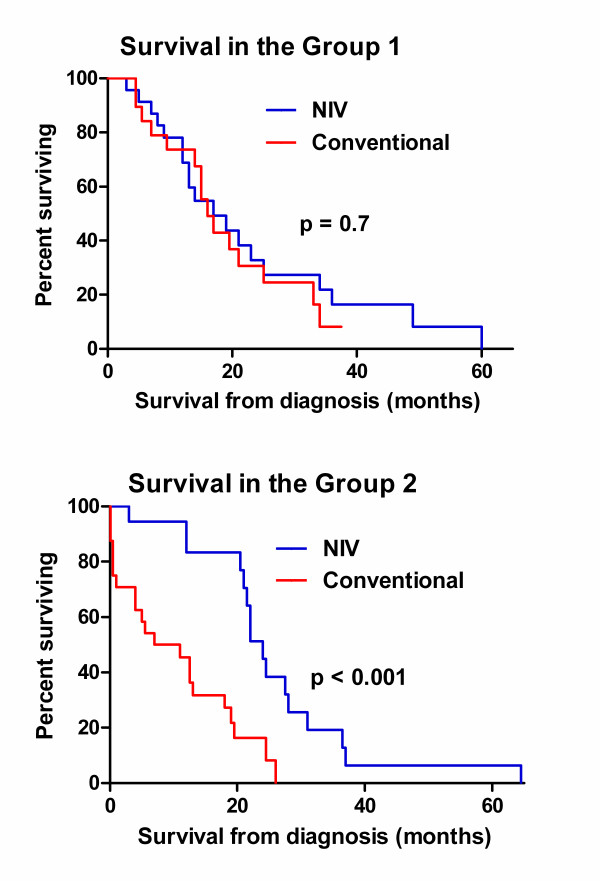
**Kaplan-Meier survival curves for patients with and without non-invasive ventilation in two age groups.** Curve comparisons were analyzed using the log rank test.

Median survival in the Group 1 NIV subgroup was 14 months (range 1 – 60) and 15 months (range 5 – 38) in the Group 1 conventional subgroup. No significant difference was found in survival between the NIV and conventional treatment groups among patients in Group 1 (Hazard Ratio = 0.88, 95% CI 0.44 – 1.77, p = 0.7). In Group 2, NIV users survived longer (median 22 months, range 3 – 65) than those undergoing conventional (median 8 months, range 1 – 26 months) treatment (Hazard Ratio = 0.25, 95% CI 0.11 – 0.55, p <0.001). Group 2 NIV non-users showed a 4-fold increased risk of mortality compared with NIV users. The Kaplan-Meier curves for both groups are presented in Figure 2.

## Discussion

This study’s retrospective analysis found that NIV use was associated with an improved survival rate in ALS patients older than 65 years. In an earlier study, del Aguila et al. found that mean age of 65 years at the time of diagnosis was an independent risk factor for adverse outcomes [3]. In the present study the risk of mortality among patients in Group 1 who did not receive NIV was four-fold when compared with NIV users. Surprisingly, there was no difference in survival rates among ALS patients under 65 years with or without NIV therapy. The median period from the onset of symptoms to diagnosis was 12 months in all four groups, indicating that there were no differences in diagnostic delay between the groups. The study’s retrospective design did not allow us to evaluate the impact of NIV on quality of life. However, 14 or more hours of NIV daily is likely to indicate compliance in both age groups.

The mechanism by which NIV may modify survival outcomes has not been fully elucidated. It is suggested that the survival outcomes in ALS patients are improved if NIV is initiated before ventilatory function is severely reduced (i.e. before the vital capacity is reduced) [12]. A prospective study conducted by Lecthzin et al. found that while thoracic compliance is reduced in ALS patients presenting with hypoventilation, inspiratory pressure support can improve compliance [16]. Based on this finding they suggested that NIV may provide nocturnal rest for fatigued respiratory muscles, thereby increasing survival rates by improving daytime functioning of respiratory muscles [12]. However, other studies considering the impact of NIV on ALS patients were retrospective in nature [12,17] and therefore their conclusions as to possible mechanisms by which NIV might improve survival outcomes are unconfirmed.

This study has some limitations. Its analysis was performed retrospectively in a relatively small patient population. The mechanisms by which NIV improved survival outcomes in Group 2 are unclear. Theoretically, improved survival may have been at least partly due to slight but statistically insignificant difference in the frequency of bulbar symptoms between the NIV users and non-users. There is compelling evidence demonstrating that survival is poorest in ALS patients with severe bulbar symptoms [10]. Poor survival may also be due to poor compliance with NIV therapy. This likely to be a contributing factor also in the present study because many Group 2 patients with bulbar dysfunction did not tolerate NIV. The results of this study should therefore be confirmed by studying a larger patient cohort and, using a prospective study design. However, because of the fact that NIV has been established as a palliative method of choice for ALS, the ethical considerations for withholding treatment should be taken seriously into account in any future prospective studies.

Second, all patients were referred for evaluation after a diagnosis of ALS was confirmed. It was therefore not possible to assess the ventilatory function of patients in the early stage of the disease, preventing us from assessing the possible impact of early NIV initiation on survival outcomes. In addition, most of this study’s patients gave consent for NIV trial in at a later stage of the disease and presented a pCO_2_ greater than 6.0 kPa, even if NIV was recommended for these patients in line with current guidelines [4-7]. It is therefore possible that this study failed to show that the initiation of NIV would have had a beneficial effect on the survival in younger NIV patients because the initiation of NIV was decided by the patient.

Factors other than NIV may also have had an effect on patient survival. These include treatment with PEG or riluzole, and gender. In addition, the incidence of hypertension and other cardiovascular diseases, decline in pulmonary function as well and whole body function may have impacted patients’ survival outcomes. PEG use may increase survival by months [18]. The same is true also for riluzole, the efficacy of which has been demonstrated in randomized controlled trials [8,9]. Female gender has been found to be an independent risk factor for worse outcomes [3]. In this study, the groups did not differ significantly in terms of gender or access to PEG or riluzole treatment. Patient numbers were low, however, resulting in a lack of power to detect significant differences between groups. It was therefore not possible to evaluate the possible effect of these factors on survival. Further studies in larger patient populations are needed to determine which factors affect clinical outcomes in ALS.

## Conclusion

The results of this retrospective cohort study suggest that the effect of NIV on survival in ALS patients is age-dependent. Use of NIV was associated with improved survival outcomes in ALS patients older than 65 years. However, further studies using a prospective design are needed to confirm the present results.

## Abbreviations

ALS: Amyotrophic lateral sclerosis; NIV: Non-invasive ventilation; FVC: Forced vital capacity; PCF: Peak cough flow; MIP: Maximum inspiratory mouth pressure; MEP: Maximum expiratory mouth pressure; SNP: Sniff nasal pressure; PEG: Percutaneous endoscopic gastrostomy.

## Competing interests

The authors declare that they have no competing interests.

## Authors’ contributions

WS, TS, AV, KO and RA designed the study. WS, TS and AV collected the data. WS, AV and TS were responsible for the interpretation of the data. WS, AV and TS prepared the manuscript. All authors participated in critical revision of the content of the article, and approved the final version.

## Pre-publication history

The pre-publication history for this paper can be accessed here:

http://www.biomedcentral.com/1472-684X/12/23/prepub
